# Opportunistic screening for skin cancer using a mobile unit in Brazil

**DOI:** 10.1186/1471-5945-11-12

**Published:** 2011-06-06

**Authors:** Edmundo C Mauad, Thiago B Silva, Maria RDO Latorre, René AC Vieira, Raphael L Haikel, Vinicius L Vazquez, Adhemar Longatto-Filho

**Affiliations:** 1 Barretos Cancer Hospital - Pio XII Foundation, São Paulo, Brazil; 2 Faculty of Public Health of University of Sao Paulo, São Paulo, Brazil; 3 Laboratory of Medical Investigation (LIM) 14 of Department of Pathology of Medical School of Sao Paulo University, Sao Paulo, Brazil; 4 Life and Health Sciences Research Institute (ICVS), School of Sciences, University of Minho, Braga, Portugal

## Abstract

**Background:**

Skin cancer is the most common malignancy in the white population worldwide. In Brazil, the National Cancer Institute (INCA) estimates that in 2010 there will be 119,780 and 5,930 new cases of non-melanoma skin cancer and melanoma, respectively. The aim of this study was to evaluate the use of a mobile unit in the diagnosis and treatment of skin cancer in several poor regions of Brazil.

**Methods:**

The diagnosis of skin cancer was accomplished through active medical screening in the prevention Mobile Unit (MU) of Barretos Cancer Hospital (BCH). The study population consisted of patients examined in the MU between 2004 and 2007, and their suspicious lesions were subjected to histopathological evaluation. Data were collected prospectively from standardized forms and analyzed.

**Results:**

During the screening, 17,857 consultations were carried out. A total of 2012 (11.2%) cases of skin cancer were diagnosed. The predominant histological type reported was basal cell carcinoma (n = 1,642 or 81.6%), followed by squamous cell carcinoma (n = 303 or 15.1%), Bowen's disease (n = 25 or 1.2%), malignant melanoma (n = 23 or 1.1%), basosquamous cell carcinoma (n = 3 or 0.1%), miscellaneous lesions (12 or 0.6%), and metatypical carcinoma (n = 4 or 0.2%). Only 0.6% of lesions were stage III. There were no stage IV non-melanoma skin lesions, as well as no melanomas stages III and IV, found.

**Conclusions:**

It was observed that the MU can be a useful tool for early skin cancer diagnosis and treatment. This program probably is important, especially in developing countries with inadequate public health systems and social inequality.

## Background

Skin cancer is the most common malignancy in the white population worldwide[1]. The 2 most common histopathological variants are basal cell carcinoma (BCC) and squamous cell carcinoma (SCC); despite their low mortality rates, these tumors can induce serious sequelae as a consequence of surgery[1]. The third most common skin cancer type, malignant melanoma, has a more aggressive behavior and consequently a poorer prognosis; malignant melanoma accounts for approximately 75% of all deaths from skin cancer[2].

There is strong evidence to date that whole-body clinical skin examination reduces the incidence of thick melanoma and, consequently, screening would reduce melanoma mortality[3]. Melanoma thickness at presentation is significantly associated with educational level[4]. In Brazil, the National Cancer Institute (INCA) estimates that in 2010 there will be 119,780 and 5,930 new cases of non-melanoma skin cancer and melanoma, respectively, which corresponds to 25% of all cancers for that period. The median 5-year survival rate for patients with melanoma is estimated at 73% in developed countries; however, it is only 56% in developing countries[5], reflecting their delayed diagnosis. Although the mortality from non-melanoma skin cancer is very low[5], important deformations can be consequences of the treatment.

The Brazilian Ministry of Health encourages the Brazilian population to avoid the sun during some periods of the day and to carefully examine their skin[5]. Few studies have reported the use of a mobile unit in the primary prevention of skin cancer[6,7]. Therefore, we did not find evidence of the use of a mobile unit equipped with a surgical room for the treatment of skin lesions. The aim of this study was to evaluate the use of a mobile unit in the diagnosis and treatment of skin cancer in several regions of Brazil.

## Methods

The diagnosis of skin cancer was accomplished through active medical search in the prevention Mobile Unit (MU) of Barretos Cancer Hospital (BCH). The study population consisted of 17,857 pre-selected patients with suspected cancer of the skin who were examined in the MU between 2004 and 2007 to confirm the diagnosis.

The trailer portion of the MU was adapted and equipped for clinical and surgical procedures. The design of the truckers' trailer accommodations and facilities was totally developed by a BCH group. The mobile unit team is composed of a clinical or surgical oncologist, a nurse, 3 nursing assistants, and a driver, and this team is able to daily perform 40 physical examinations of the skin (with surgery and cryotherapy). In this study, we used the MU only for skin cancer prevention, diagnosis, and treatment. The MU is intended to travel to small cities and rural areas in several States of Brazil, including the Amazonas region. The MU visit is normally preceded by a phone call from a health care professional from the Department of Prevention of BCH; this individual makes the first contact with local authorities of the cities to be visited and plans the training of nurses and social assistants from these cities in the BCH. These local nurses are trained all day to identify suspicious lesions for skin cancer by the nurses and a physician from Barretos Cancer Hospital with experience in skin cancer screening. Specific conferences are offered in order to exemplify the different types of the more frequent skin lesions, and didactic materials (e.g., images CD, banners, leaflets and brochures) are given to support the educational training. These nurses are also responsible for speaking with the clinicians in these cities to convince them about the importance of their participation in the prevention activities and also for closely assisting the nurses' triage. In addition, they are encouraged to principally invite the underprivileged people that live in the suburbs or in rural areas and to inform them that all of the procedures related to the cancer prevention and treatment are free of charge.

Patients examined in this study were evaluated in the MU, and their suspicious lesions were subjected to histopathological evaluation, local resection with margins, or local resection with skin flaps or use of a skin graft. Patients with large lesions or patients with a need for complex surgical procedures were scheduled to be examined and treated at BCH. The samples collected were properly identified and forwarded to the Pathology Department of BCH, where reports were ascertained. Precursor lesions were treated with cryotherapy in the MU.

One copy of the histopathological results was mailed to each patient and another to the cities' nurses involved in this project. A secretary in the Prevention Department of BCH scheduled a consultation by telephone. The patients with a skin cancer diagnosis who only underwent biopsy or had involvement of the surgical margins were followed to radiotherapy or surgery at the BCH. The patients who did not respond to the call for BCH treatment received a second invitation. If the patients did not respond to this new invitation, a third letter was sent to the patient and simultaneously to the public health authorities of the municipality, informing them of the need to do a medical consultation in another Institution.

Data from patients cared for in the MU were collected prospectively from standardized forms at the Department of Prevention and recorded. When a case of skin cancer was diagnosed, the information was sent to the Hospital Cancer Registry of BCH. The variables evaluated were as follows: gender, local of residence, year of first attendance, age, schooling, pathologic diagnosis, and stage[8]. The number of lesions excised, the sizes of the lesions, the location of the major lesion, the type of surgery, and the type of additional therapy (surgery or radiotherapy) were analyzed only for the MU patients who underwent surgery.

Descriptive analysis was performed using frequencies and percentages, as well as measures of central tendency and dispersion (mean, median, minimum and maximum values).

The clinical and pathological data were collected from the medical records of the patients. Data were stored and statistical indexes calculated in a database set up in the software SPSS for Windows 17.0 (Inc., Chicago, IL, USA).

The Ethics Committee of the Barretos Cancer Hospital approved this study protocol. All the Consent informed terms were signed and are currently archived in Prevention Cancer Unit under supervision of the local Ethical Committee.

## Results

During the 4 years of skin lesion screening, 17,857 consultations were carried out and 3,005 biopsies or surgeries were performed. From this, a total of 2012 (11.2%) cases of skin cancer were diagnosed in a slight majority of women (51.1%); 85.6% of the patients were aged 40-79 years (Table 1). The educational level of the patients was predominantly low: 87.5% were illiterate or had completed 8 years of basic study, 7.7% had completed between 8 and 12 years of study, and 4.8% had incomplete or complete higher education.

**Table 1:** Case distribution of non-melanoma and melanoma skin cancers in patients served by the Mobile Unit

Variable	Category	Screening n° (%)
Gender	Male	983 (48.9)
	Female	1,029 (51.1)
Age (years)	< 40	92 (4.6)
	40 - 59	625 (31.1)
	60 - 69	573 (28.5)
	70 - 79	525 (26.1)
	≥ 80	197 (9.8)
Origin state	São Paulo	1,086 (53.7)
	Mato Grosso	500 (24.9)
	Mato Grosso Sul	195 (9.7)
	Rondônia	94 (4.7)
	Minas Gerais	84 (4.2)
	Goiás	53 (2.6)
Clinical Stage	0	40 (2.0)
	I	1840 (91.5)
	II	110 (5.5)
	III	13 (0.6)
	IV	0 (0)
	X	9 (0.4)
Diagnosis	BCC	1,642 (81.6)
	SCC	303 (15.1)
	Melanoma	23 (1.1)
	Metatypical Cancer	4 (0.2)
	Other	12 (0.6)
	Bowen's disease	25 (1.2)
	Basosquamous CC	3 (0.1)

Total		2,012

The majority of the patients (82%) with skin cancer had resection of the lesion performed in the MU, and 74.6% had only one suspicious lesion that was surgically removed. The remaining patients had 2 or more skin lesions. Such lesions were detected mostly in the head and neck region (78.7%). Lesions on the trunk, upper limbs, and lower limbs appeared in 12.7%, 7.8%, and 0.8% of cases, respectively.

Of the total number of diagnosed patients who had screening procedures performed in the MU, only a biopsy was performed in 230 (11.4%) of the patients. The remaining 132 (6.6%) patients who had no surgical procedure performed in the MU were sent to BCH for reasons such as a high-risk medical condition, advanced age, a very large lesion for which biopsy is not indicated, or a preference for treatment in our hospital. Unfortunately, of these 230 patients, 17.9% did not respond to the call for treatment. The biopsy procedure was used for large lesions, for those that would cause difficulty in reconstruction, for lesions in cartilage, or for lesions in patients submitted to adjuvant radiotherapy. For 1,113 (55.3%) lesions, we performed resection and simple suture; for 645 (32.1%) lesions, we performed resection with flap rotation; and for 24 (1.2%) lesions, we performed resection and skin grafting. Of the 3,005 suspicious lesions analyzed, 67% showed evidence of cancer on histopathological examination, and the predominant histological type reported was basal cell carcinoma (n = 1,642 or 81.6%) (Table 1). Our findings show that the sensitivity rate (cases of skin cancer per suspicious lesions analyzed) ranged from 0% to 61% in the cities visited.

The clinical stage or the stage of cancer development (amount or spread of cancer in the body) of the lesions was evaluated. The vast majority of non-melanoma skin cancers (93.9%) were stages 0 and I; 5.5% of cancers were stage II and 0.6% of cancers were stage III. For melanomas, 88.8% were stages 0 and I and 11.2% were stage II. The median thickness of these lesions was 0.9 mm (range: 0.5 to 4.0 mm). There were no stage IV non-melanoma skin lesions, as well as no melanoma stages III and IV, found.

## Discussion

Early diagnosis of skin cancer is crucial for obtaining the best treatment results, improving the prognosis, and, most importantly, increasing the quality of life of the patient. However, there is no robust evidence to ascertain whether there is a decrease in mortality due to regular physical examination of the skin[7]. Considering, e.g., melanoma, the diagnosis of incipient lesions, up to 1.00 mm thick, provides an excellent survival rate of 97% after 5 years[9]; but the survival rate is only 7% to 19% if stage IV lesions are found[10]. More recent data show that the one-year survival rate is 33% in patients with metastatic melanomas (stage IV) [9]. Conversely, and despite the low rates of mortality and low potential for metastasis, early diagnosis of non-melanoma skin cancer is also essential to reducing the cost of treatment and the associated consequences[11,12].

Importantly, we observed in this study a large proportion of lesions of more than 2.0 cm in diameter among the non-melanoma cancers (129 or 6.5%), as well as melanomas that were 0.75 mm to 1.5 mm in thickness (3 or 13.0%). This may reflect, in part, the lack of public health cancer prevention programs, which creates many barriers to medical evaluation and care. The *Fundação Oncocentro de São Paulo - FOSP *(São Paulo Cancer Foundation) is a Government Foundation with a database of the hospital cancer registry of São Paulo State. The records show that during the same time period (2004 to 2007), 9.4% of non-melanoma skin cancers were more than 2.0 cm in diameter[13]. Although there is a concerted effort to provide good health services equally to all citizens, this is not the reality in this country. This is because there are regional and interstate differences in health care.

Use of a mobile unit for the early diagnosis of other cancers, such as breast and cervical cancer, has been used for decades in many countries[14] and has shown good results in remote and rural Brazilian areas[6,7,15-17]. This strategy is still being used for breast cancer screening in developed countries[18]. The UMP of BCH is the exclusive initiative to "catch up" patients in remote areas in Brazil. To our knowledge, it is also unique anywhere in the world to conduct small and medium skin surgeries in an MU. Few reports of the use of an MU for skin cancer screening are found in developed countries, probably due to a more well-developed health care structure and a population with better information on means of preventing this cancer[5,19]. Since changes in the health care system are usually slow, the use of an MU provides faster specialized medical care to the people with difficult access to health services. At the same time, it provides the opportunity to disseminate information about the importance of cancer prevention. One good example of MU use in the industrial field is the Senai Mobile Technical School. This Brazilian government department developed programs to prepare manpower in locations not served by fixed schools. Its structure of mobile units allows for more flexibility in attendance and a greater exchange between the institution and local industries [20].

Even with little training time with the staff of the Department of Prevention, the nurses in municipalities have obtained reasonable results in the screening of suspicious lesions, with 2,012 positive cases in 17,857 assessments (11.3%).

The training of health care professionals is of paramount importance to the project. Possibly, teledermatology is a feasible option for improving the project, because of the huge distances in remote areas visited by the MU[21-23]. We have also observed a great commitment from the poorest populations of the cities visited, especially in the Amazon region, where 73.9% of people are illiterate or only have up to 8 years of schooling. This commitment to the MU is a very welcomed situation, but it also indicates the lack of an adequate system of public health in municipalities and states. In many cities, people have anxiously remained waiting in close proximity to the MU since very early in the morning to be sure that a doctor will examine them. Sometimes, they have stayed until almost midnight because they are afraid that the MU would not remain another day in that city.

In Brazil, 70% of the cities have less than 20,000 inhabitants, and approximately 20 million inhabitants still live in rural areas[24]. Dermatologists or general practitioners with appropriate experience to identify early skin cancer lesions are most likely not found in these areas, explaining the reduction in cases of skin cancer diagnosis and treatment in these populations. According to the Brazilian Society of Dermatology, the vast majority of dermatologists are in São Paulo State (32.8%). Some States visited by the MU have only 0.1% of Brazilian dermatologists; this means that there is only 1 dermatologist for 306,609 inhabitants[25].

The skill of a well-trained medical oncologist prepared to identify skin lesions and perform the surgical incisions was well exemplified in our study: 67% of the lesions surgically removed were categorized as cancer, and importantly, 82% of the patients with skin cancer had resection of the lesion at the MU. The false positive rate was 33%, once 3,005 surgeries were performed..

Skin cancer prevention is cost-effective[26,27]. This is an important issue to be discussed with governmental public health authorities to improve the detection of early stages of skin cancer. Potential barriers to hamper the efficacy of skin cancer screening include the reduced time to perform a screening during a regular physical examination and the lack of a specific training program for dermatologists to recognize lesions that are potentially neoplastic[28]. In Brazil, only 74.1% of dermatologists completed a fellowship or have a specialist title[25]. Health care professionals, in general, do not counsel patients regarding the prevention of skin cancer except for dermatologists[29].

The Barretos Cancer Hospital overall mobile unit (MU) cost is $566,181.17 USD. This cost represents several components of the MU as follows: tractor truck $205,882.35 USD; semi-trailer $58,823.53 USD; semi-trailer modifications $235,294.11 USD; equipments $58,823.53 USD, and marketing adhesives $7,357.65 USD. To carry out 800 to 1.000 monthly skin lesions evaluations, MU have additional monthly costs with professionals salary and taxes: one physician ($11,764.70 USD), one nurse ($2,115.32 USD), two nurse's assistants ($864.85 USD), one typist ($630.00 USD) and 2 MU drivers ($932.56 USD), resulting in an annual cost of $217,258.52 USD.

Consumables costs for MU travel maintenance correspond to $8,000.00 USD per month: fuel ($2,941.18 USD), toll ($352.94 USD), mechanic vehicle maintenance ($4,705.88 USD). For staff's accommodation and meal during the travel and the monthly cost is $882.35 USD. All included, the monthly operational cost is about $16,882.35 USD.

Public Health System fees for consultations/examinations, biopsies and resection, are $5.88 USD, $8.30 USD and $7.33 USD, respectively. The numbers of procedures for the 2010 were 800 attendances monthly which correspond to 800 consultations/examinations ($4,705.88 per USD month), 172 biopsies ($1,429.92 USD per month) and 24 resections ($179.02 USD per month). The total annual income was $75,777.88 USD.

Notice however that the same mobile unit that performs the screening of skin cancer, also, performs screening for cervical cancer (Pap smear) and prostate cancer, attenuating the total costs.

Despite the high exposure to the solar rays, the Brazilian population has lower incidences of melanoma and non-melanoma skin cancer compared to the Australian population[27]. A speculative reason for this difference could be the high miscegenation of the Brazilian population, which includes indigenous individuals and African descendants, who live in these states and create protective characteristics against skin cancer for many people. The other European descendants choose to live in the south or southeast of Brazil, where the weather is colder and there is less sun exposure than in other regions of the country.

Unfortunately, we failed to encourage the participation of local medical doctors in these efforts toward cancer prevention. Alternatively, our strategy of having those physicians invited by a nurse to participate in this project may not be the most adequate recruitment strategy.

The lack of medical oncologists and dermatologists in the visited cities, especially in smaller towns and rural areas, was a major limitation of this project. This is probably due to the reduced value of medical remuneration paid by governmental public health authorities, which clearly encourages these professionals to work preferably at private clinical services. This lack of doctors makes it difficult to supervise the nurses' triage of the patients.

Another limitation was the limited experience of nurses in some municipalities. The rate of 11.3% of positive cases among patients invited by nurses to undergo screening ranged considerably. This is mainly due to the short training and lack of time some nurses in those municipalities had to work on this project. To improve the quality of patient selection in each city and to increase the rate of cancer patients examined for lesions, the training of nurses should gain more attention from the staff of the Barretos Cancer Hospital. The lessons will be refurbished and the supplied material will be restructured. These nurses will also be invited to monitor the operations performed by the mobile unit, which may include following the procedures and consultations with a specialist.

Is important to say that this is a pre-selected population, so these results cannot be established on the prevalence or distribution rates of skin cancers in the Brazilian general population.

Despite these limitations, we were able to diagnose 2012 cases of skin cancer and more than 80% of these patients were operated on in the MU.

## Conclusions

It was observed that the MU can be a useful tool for the screening and treatment of skin lesions, providing a quick and effective solution for reaching populations in remote areas, rural municipalities, or regions with low access to a public health system. This is an effective method of underscoring the necessity to improve assistance to underprivileged populations to minimize the occurrence of advanced cases of skin cancer in a country with an inadequate health care system.

## Competing interests

The authors declare that they have no competing interests.

## Authors' contributions

ECM was involved in drafting the manuscript and revising it critically with important intellectual content. TBS carried out the collection of data and drafted the manuscript. MRDOL participated in the design of the study and the interpretation of data. RACV was involved in drafting the manuscript and revising it. ALF participated in the design of the study and revising the manuscript critically with important intellectual content. RLHJr participated in the skin cancer surgeries and critical revising of the manuscript. VLV contributed to critically revising the revised version of the manuscript. All authors read and approved the final manuscript.

## Pre-publication history

The pre-publication history for this paper can be accessed here:

http://www.biomedcentral.com/1471-5945/11/12/prepub
